# Persister-mediated emergence of antimicrobial resistance in agriculture due to antibiotic growth promoters

**DOI:** 10.3934/microbiol.2023038

**Published:** 2023-11-13

**Authors:** Noah T Thompson, David A Kitzenberg, Daniel J Kao

**Affiliations:** 1 Department of Medicine and Mucosal Inflammation Program, University of Colorado Anschutz Medical Campus, Aurora, Colorado, USA; 2 Medical Scientist Training Program, University of Colorado Anschutz Medical Campus, Aurora, Colorado, USA

**Keywords:** resistance, agriculture, persistence, tolerance, antibiotics, catalyze, bacteria, antimicrobial resistance, antibiotic growth promoters, subtherapeutic

## Abstract

The creation and continued development of antibiotics have revolutionized human health and disease for the past century. The emergence of antimicrobial resistance represents a major threat to human health, and practices that contribute to the development of this threat need to be addressed. Since the 1950s, antibiotics have been used in low doses to increase growth and decrease the feed requirement of animal-derived food sources. A consequence of this practice is the accelerated emergence of antimicrobial resistance that can influence human health through its distribution via animal food products. In the laboratory setting, sublethal doses of antibiotics promote the expansion of bacterial persister populations, a low energy, low metabolism phenotype characterized broadly by antibiotic tolerance. Furthermore, the induction of persister bacteria has been positively correlated with an increased emergence of antibiotic-resistant strains. This body of evidence suggests that the use of antibiotics in agriculture at subtherapeutic levels is actively catalyzing the emergence of antimicrobial-resistant bacteria through the expansion of bacterial persister populations, which is potentially leading to increased infections in humans and decreased antibiotic potency. There is an urgent need to address this debilitating effect on antibiotics and its influence on human health. In this review, we summarize the recent literature on the topic of emerging antimicrobial resistance and its association with bacterial persister populations.

## 1. Importance and use of antibiotics in agricultural and clinical settings

### 1.1. Prevalence of antibiotics: discovery and resistance

The discovery and implementation of antibiotics is one of the greatest medical and scientific triumphs in all human history. Prior to their discovery, people, plants and animals were left defenseless against now-trivial infections. Most of the antibiotics currently in use were developed during the 20^th^ century, and their impact cannot be overstated. The estimated extension to the human lifespan is approximately 23 years after the introduction of antibiotics [1].

The impact of antibiotics on plants and animals has also been substantial, particularly in agriculture, which represents the majority of global antibiotic usage [2]. It has been reported that approximately 93,000 tons of antimicrobials were sold in 2017 [3]. In the next decade, antibiotic sales are projected to reach 104,000 tons [3]. Antibiotic consumption practices vary widely based on sector (healthcare vs. agriculture), geography and country income status [2],[4]. In human health sectors, antibiotics are primarily used to treat existing infections, as precautionary prophylaxis during surgery or for patients with compromised immunity. In animals, beyond veterinary infection treatment, antibiotics are often used as nontherapeutic growth promoters to increase animal food production to help keep pace with the increasing demand for animal protein or prophylactically against infection where hygiene practices are poor [5]. Despite these myriad benefits of antibiotics, their uses are not without consequences. Antibiotic resistance, which falls under the umbrella of antimicrobial resistance (AMR), is a growing global health threat, and inappropriate antibiotic use in animals is a major driver of AMR [5]. This is a significant problem because many antibiotics that are used in animals are also used in humans, including important quinolones, beta-lactams, aminoglycosides, tetracyclines, macrolides and other antibiotic classes [6],[7]. Even though antibiotic usage practices differ between healthcare and agriculture, a complex AMR dynamic has formed between the two sectors due to the similarity of antibiotic usage, where antibiotic resistance can emerge in either setting and have profound impacts on both agriculture and human health.

AMR already poses a significant global health burden. In 2019, roughly 5 million deaths were associated with AMR and 1.27 million deaths were attributable to AMR [8]. Antibiotic-resistant bacteria are a significant and growing threat across healthcare, veterinary and agricultural settings, with the possibility of untreatable infections becoming an increasing reality [9],[10]. Understanding the mechanisms of resistance emergence and acquisition is critical for counteracting these increasingly difficult-to-treat pathogens and preserving the efficacy of current antibiotics.

To better understand AMR, researchers have looked towards resistance acquisition. Growing evidence suggests that two bacterial traits, antibiotic tolerance and antibiotic persistence, may play important roles in the bacterial development and acquisition of antibiotic resistance [11]–[14]. Antibiotic tolerance and persistence were first described in the early part of the 20th century, shortly after the discovery of penicillin [13]–[15]. However, over the last couple of decades, technological advances in microfluidics, imaging and a variety of molecular biology techniques have reinvigorated research into these two traits [16],[17]. This renewed interest has rapidly expanded our understanding of tolerance and persistence, while also highlighting their complex interplay with resistance. Due to the diverse nature of infections, host and environmental factors, antibiotic mechanisms of action and resistance mechanisms, as well as the wide range of antibiotic usage amongst humans, plants and animals, antibiotic tolerance and persistence research will remain a fast-growing and rapidly evolving field. Additionally, a better understanding of bacterial resistance and its interaction with animals and humans will help inform health authorities concerning the steps of the food production pipeline. This review sets out to examine our current understanding of antibiotic tolerance and persistence in the context of antibiotic use in agriculture. Additionally, it proposes a novel model for viewing AMR emergence and presents possible countermeasures to combat this emergence.

### 1.2. Discovery and use of antibiotic growth promoters

In 1946, a research group at the University of Wisconsin discovered that chicken feed supplemented with two different antibiotics–sulfasuxidine and streptomycin–led to an increase in growth [18]. This discovery triggered several other groups to confirm these findings and broaden the scope of the effect to other antimicrobial compounds and other species of livestock. A similar phenomenon was discovered in swine and poultry sectors, classifying these compounds as “antibiotic growth promoters” (AGPs). The use of antibiotics caused a 1–10% increase in the daily growth rate of livestock compared to nontreated groups. Additionally, pigs that were fed antibiotics with their feed, required 10–15% less feed to reach a benchmark body weight [19]. These findings were readily implemented, leading to a global increase in the use of AGPs. As of 2015, it has been observed that 50–80% of all manufactured antibiotics are used as AGPs with China, Brazil and the United States being attributed with the highest consumption rates prior to certain AGP bans that will be discussed in brief further on in this review [2],[3],[20],[21].

There are multiple proposed mechanisms of AGPs, each with its own merit. The first three mechanisms relate to a global decrease in the gastrointestinal microbiota of the host, namely (1) a decrease in bacterial consumption of nutrients, leading to an increase of nutrient availability by the host; (2) decreased bacterial metabolites that are inhibitory to host growth and (3) inhibition of inapparent infections within the host [22]. The fourth proposed mechanism of action for AGPs is that livestock that are fed AGPs develop thinner intestinal walls, and thus, it is speculated they can more readily uptake luminal nutrients [22]. However, the aforementioned mechanisms are repudiated by Niewold and an alternative is presented [23]. Niewold argues that the anti-inflammatory nature of certain classes of antibiotics is a more likely mechanism for AGP function–even though inconsistencies exist with this theory–than the antimicrobial nature of these compounds. Niewold's hypothesis appears to fit most within the cycline, macrolide and streptogramin classes of antibiotics because all three classes tend to inhibit phagocytic functions and are used as AGPs [23]. While his postulations seem convincing, Niewold fails to address two key counterpoints: (1) the phenomenon that AGPs tend to have minimal effect on germ-free animals [24] and (2) the fact that antibiotics given at doses below the minimum inhibitory concentration do produce other modulatory effects on certain microorganisms [25] which could potentially lead to dysbiosis by changing the competitive landscape of the intestine. In addition, antibiotics that are not readily taken up from the intestine–bacitracin and streptomycin–and produce a growth-induced phenotype when supplemented in the diet show minimal impact on livestock growth [26]. Moreover, chloramphenicol–an antibiotic that is readily absorbed in the stomach but interacts minimally with the intestine–produces minimal growth effects on livestock despite its potent antimicrobial activity, highlighting the importance of the growth phenotype on the interaction between the AGP and the intestine [27].

Thus, despite such widespread use of AGPs, the exact mechanisms remain controversial. With the rapid expansion of investigation into the microbiome, more recent research on AGPs has revolved around the gastrointestinal microbiota [28]–[31]. Ultimately, the mechanisms of AGPs are likely a combination of the antimicrobial and antimicrobial-independent activities and other currently undiscovered effects of these compounds.

### 1.3. Dissemination of AMR from agriculture to humans

Regardless of the mechanism of growth promotion, there is little doubt that antibiotic use in agricultural food production supports the *de novo* development of AMR and drives the colonization of agricultural animals by resistant organisms [7],[32]–[38]. On the other hand, it has been surprisingly difficult to conclusively demonstrate that the use of AGPs directly drives antimicrobial resistance in human pathogens. A compelling body of evidence in the study of gastroenteritis caused by fluoroquinolone (FQ) resistant *Campylobacter* bacteria supports the hypothesis that antibiotic use in agricultural food production leads to human infections with resistant microorganisms. *Campylobacter spp*. can be considered a nexus between the microbiology of agricultural food production and human infectious disease. *Campylobacter* species commonly cause enteric bacterial infection in humans, and exposure is generally from raw or undercooked meat products. They are commensal organisms colonizing the avian gastrointestinal (GI) tract, particularly poultry used for food production, but are not considered commensals of the human GI tract. Human-to-human infection by *Campylobacter spp*. is also considered rare. Because of this unidirectional transmission pattern, *Campylobacter spp*. are particularly relevant to studying the spread of antibiotic resistance from agricultural food products to humans.

Several studies have investigated whether the use of AGPs contributes to antibiotic-resistant *Campylobacter* infections in humans. A particular focus of these studies has been the rate of FQ resistance before and after the licensure of FQs for veterinary use. The first generation of quinolone antibiotics was developed in the late 1960s. The second and third generation quinolone antibiotics, which were the first fluoroquinolones (FQ), were approved for human use in the mid-1980s and quickly went into widespread use, e.g. ciprofloxacin was approved for clinical use in the United States in 1987 [39]. In most countries, approval for veterinary use did not occur until the early 1990s, though quinolones were approved for veterinary use in some countries in the 1980s. This time gap between the start of widespread human use and widespread veterinary use has been the focus of much scrutiny into the influence of AGP use on AMR in a human pathogen. The key theme of these studies has been that FQ resistance was rare prior to 1989 and many studies have temporally correlated the rise of the prevalence of resistance to the approval of FQs for animal use.

There have been several studies that link veterinary antibiotic use to the increased prevalence of AMR. Furthermore, increased use directly correlates with increased rates of AMR [40]. In the context of this review, of particular interest is how AMR in agriculture influences human disease. In 1986, Spain was one of the first European countries to authorize quinolone use in cattle, pigs and poultry. A study published in 2000 concerning *Campylobacter spp*. resistance to ciprofloxacin revealed that 99% of *Campylobacter* isolates from agricultural animals and animal food products, as well as 72% of *Campylobacter* isolates from infected humans, were resistant to ciprofloxacin. In contrast, Norway has traditionally enforced more restrictive policies regarding veterinary antimicrobial use compared to other European countries such as Spain. Norway also maintains a comprehensive system for monitoring antimicrobial resistance in wild and domestic animals. A study published in 2006 showed that the prevalence of FQ-resistant *Campylobacter* isolates from Norwegian broiler chickens was low (1–3%) during the study period of 2001 to 2003, a stark contrast to the Spanish figures at an earlier time point. Likewise, the prevalence of FQ-resistant *Campylobacter* from humans who had not traveled outside of Norway was relatively low (3–11%) [41]. In contrast, the prevalence of FQ resistance in *Campylobacter* isolates from individuals who were thought to have acquired their infections from outside of Norway was much higher (59–73%). Assuming that animal food products are primarily distributed within the country of origin, these data suggest that AMR patterns observed in isolates causing human disease mirror those observed in the locally produced animal food products. In turn, AMR rates in agricultural animals correlate with antimicrobial use policy in a given country. Based on these types of studies, many have concluded that veterinary use of antibiotics in food production contributes significantly to the infection of humans by antimicrobial-resistant microorganisms and represents a significant issue in food safety in general.

The rise of antibiotic resistance in human isolates has been correlated with rates of resistance seen in animal isolates. However, the causal linkage has been largely difficult to establish. Molecular studies have been undertaken in efforts to unequivocally make this link, and while there are numerous similarities between resistance mechanisms in strains infecting humans and agricultural animals, these efforts have generally been inconclusive [42],[43]. A systematic review performed in 2018 identified 45 studies investigating the directionality of AMR [44]. Only eight of the 45 studies came to conclusions that supported the transmission of AMR in the direction of animals to humans. A portion of the remaining studies concluded that the mechanisms of bacterial resistance in agricultural animals and humans were shared, but the direction of transmission could not be determined. The rest of the studies concluded that there was no evidence that AMR mechanisms were shared. The authors of this systematic review concluded that there was insufficient evidence to unequivocally support the hypothesis that the primary direction of AMR transmission is from agricultural animals to humans and noted that further studies employing high-resolution genomic sequencing are needed to answer this question. While controversy in this field remains, the underlying mechanisms of AMR emergence continue to be elucidated through the study of bacterial persistence, tolerance, and ultimately resistance.

## 2. Persistence, tolerance and resistance

### 2.1. Definition, mechanisms and induction of bacterial persistence

Antibiotic resistance is a well-known and well-characterized microbial phenomenon that allows bacteria to survive and grow in the presence of antibiotics. It is typically characterized by an increase in the minimum inhibitory concentration (MIC), which is the lowest concentration of an antibiotic required to prevent bacterial replication and growth. However, two other bacterial phenomena, termed antibiotic tolerance and antibiotic persistence, contribute to antibiotic treatment failure. Studies over the last few decades have elucidated the importance of tolerance and persistence in the context of antibiotic treatment and resistance [5],[11],[16],[45]–[47]. Antibiotic tolerance and persistence are phenotypic traits that allow antibiotic-susceptible bacteria (no increase in MIC) to transiently survive exposure to high concentrations of different classes of antibiotics without the bacteria possessing traditional heritable resistance to the antibiotic [17],[48]. Tolerance and persistence are often used interchangeably, but they present important differences. Therefore, it is important to define tolerance and persistence to promote a better understanding of these phenomena within different systems [48]–[50]. The definitions of antibiotic tolerance and persistence presented here are based on consensus guidelines; they have been established through experimental observations and do not define specific mechanisms of tolerance or persistence [48]–[50]. Tolerance or persistence emerges when bacteria are challenged with bactericidal antibiotics, and they are characterized by killing rates, commonly presented as the minimum duration for killing (MDK). MDK curves are log-linear plots of bacterial survival as a function of time during antibiotic exposure, and they are used to distinguish tolerant and persistent populations from antibiotic susceptible populations. The bacterial response to a bactericidal antibiotic can be broadly divided into 4 categories and can be distinguished on MDK curves: (1) Antibiotic susceptible, in which the antibiotic causes a steady rate of killing in the bacterial population; (2) Antibiotic resistant, in which bacteria do not die from treatment and can grow in the presence of the antibiotic; (3) Antibiotic tolerant, in which the antibiotic causes a steady but slower rate of killing when compared to the killing rate of an antibiotic susceptible population (presents as a shallower killing slope on an MDK curve; and (4) Antibiotic persistent, in which a large fraction of the bacterial population is antibiotic susceptible and is killed at a steady rate, while a smaller subpopulation of bacteria is antibiotic tolerant, or heterotolerant, and is killed at a slower rate, resulting in a biphasic kill curve [49],[51]. This biphasic killing is a hallmark of persister populations [48]. Almost all bacterial species examined to date can form persister cells, and they typically represent 0.001–1% of the clonal population [5]. Therefore, tolerance is a population-level trait while persistence describes a subpopulation of cells that experience reduced antibiotic killing relative to the susceptible bacteria within the population [48]. It is also important to note that isolating and culturing the tolerant or persister bacteria after an antibiotic treatment will typically result in the outgrowth of a population that remains susceptible to the antibiotic (no change in MIC), assuming resistance was not acquired in an alternative manner [49].

Antibiotic tolerance and persistence are highly dynamic processes that can occur through many different mechanisms and can be triggered by diverse environmental or experimental stimuli. Broadly, cells exhibiting these phenotypes are often thought of as transiently, nonproliferative cells with altered metabolism or energetics, changes in antibiotic uptake or reduced antibiotic target activity [5],[17],[48],[49],[52],[53]. Tolerance can be driven by genetic mutations, slow growth or delayed exit from lag phase, and it can often be triggered by an environmental stressor that impacts the entire bacterial population, such as growth-arresting starvation, exposure to a particular compound such as a bacteriostatic agent or reactive oxygen species among other stressors [49],[50],[54]–[56]. Persistence can also be triggered by environmental stressors such as reactive oxygen species, antibiotics or bacteriostatic agents, as well as phagocytosis by host cells or other diverse stimuli [50],[55],[57]–[59]. Mutations in or activation of various toxin-antitoxin systems, such as *hipA* and *hokB*, can also influence persister formation and change the fraction of persisters within a population [60]–[62]. Persistence can also occur stochastically, but it appears to occur at a lower frequency when compared to induced persistence from the activation of a bacterial stress response [50],[63]. The concentration of persister-promoting proteins may randomly fluctuate within a given bacterium, which leads to a small fraction of bacteria within a population adopting a persistent phenotype [17],[46],[64]. This is considered to be a form of biological bet-hedging [64]. In the context of growth and expansion, the overall fitness of a population may be reduced by the presence of a persister subpopulation that is dormant or non-replicative; however, this subpopulation is better able to handle various stress conditions such as rapid environmental changes or antibiotic treatment. Both methods of persistence formation, i.e., induced or stochastic, can act redundantly and influence the cellular state to promote dormancy, low activity or an active yet nonreplicative state. These changes decrease the activity of cellular processes that are targeted by antibiotics leading to decreased potency of the antibiotic towards the bacterium [5],[17],[53],[65]–[67]. Increased efforts are being directed toward understanding the diverse and complex mechanisms of tolerance and persistence, which vary widely based on bacterial species, antibiotic pressure and experimental conditions, among other crucial factors. A deeper understanding of tolerance and persistence will better inform antibiotic usage practices and may provide new therapeutic opportunities.

### 2.2. Persistence leading to antimicrobial resistance

Understanding the mechanisms that drive the emergence and spread of AMR is crucial for combatting it effectively. Early investigations into tolerance and persistence have revealed that both tolerance and persistence function as a means to acquire resistance, either vertically through de novo mutation or horizontally through resistance plasmids [11],[14],[51],[68]. A 2017 study by Levin-Reisman et al. found that when *E. coli* was cyclically challenged with the beta-lactam antibiotic ampicillin, tolerance increased and preceded the emergence of antibiotic resistance. Whole genome sequencing revealed that the canonical *ampC* mutation, which confers resistance, occurred after each strain acquired antibiotic tolerance-promoting genetic mutations. They propose that tolerance extends the opportunity for rarer, antibiotic resistance mutations to occur and enhances the establishment of antibiotic resistance mutations through the epistasis between tolerance and partial resistance to the antibiotic. The findings support that antibiotic tolerance functions as a stepping stone for the emergence of genetically heritable antibiotic resistance mutations. Another study demonstrated that persistence and the evolution of resistance are positively correlated in multiple natural and lab strains of *E. coli* through increased survival and mutation rates [14]. Moreover, another group examined the role of persisters in the emergence of de novo resistance from a single treatment of the fluoroquinolone ofloxacin, which causes DNA damage and can kill nonreplicating bacteria. When challenged with a single ofloxacin treatment, persister bacteria promoted the emergence of more antibiotic-resistant mutants through increased ofloxacin-induced DNA mutations. The recovery of ofloxacin-treated persisters also led to the enrichment of mutants that displayed resistance to multiple unrelated classes of antibiotics [51]. This demonstrated that from a single antibiotic treatment, persisters serve as a reservoir for fluoroquinolone-driven DNA mutations that can confer resistance to other types of antibiotics. To investigate the role of persisters in horizontal gene transfer of resistance, one group examined *Salmonella* in a mouse gut infection model and found that *Salmonella* persisters can survive ciprofloxacin treatment and function as long-lived reservoirs for the horizontal transfer of antibiotic resistance-conferring plasmids through conjugation [68]. This study also provides an important demonstration that persistence can influence resistance acquisition and spread in vivo. Horizontal gene transfer of antibiotic-resistant elements represents an important mode of AMR spread. So, targeting persistent populations may represent a therapeutic opportunity for reducing the spread of AMR plasmids between bacteria [69],[70]. It is also feasible that other stressful conditions, like the introduction of common disinfectants, ultra-violet light, acidic or alkaline conditions, heat or osmotic stress, may increase AMR bacteria through the persister phenotype [71],[72]. Research into resistance emergence and its spread through tolerant and persistent bacteria is still in the initial stages, yet mounting evidence supports the interdependence between the three concepts. Targeting antibiotic-tolerant and persistent bacteria represents a very appealing approach for combatting AMR and improving infection treatment. The development of antibiotic adjuvants that enhance antibiotic killing of tolerant or persistent bacteria may be an effective method for improving existing antibiotics and decreasing the emergence of microorganisms that are resistant to them [13],[52],[73]–[77]. There are alternative mechanisms of AMR emergence which have been covered extensively in other reviews [78]–[80].

## 3. Antibiotics, persistence, and resistance in agriculture

### 3.1. Novel model of AMR emergence in agriculture

**Figure 1:**
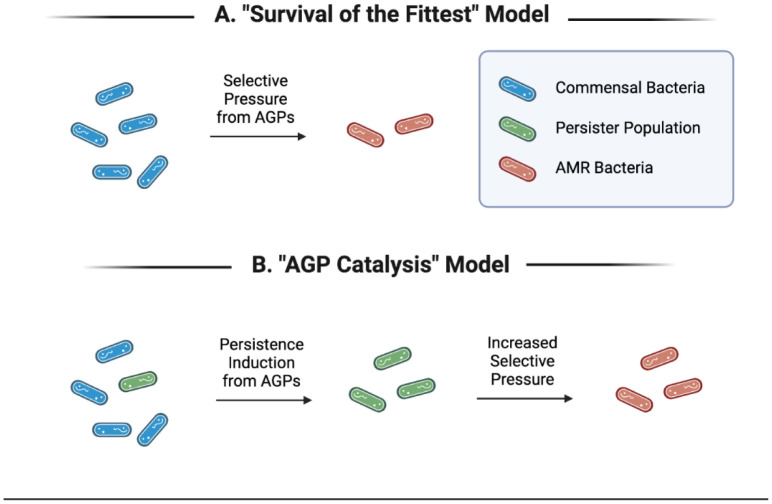
Two different models of viewing the emergence of antibiotic-resistant bacteria in agriculture. The "survival of the fittest" model (A) demonstrates the passive emergence of resistant bacteria as an innate feature of antibiotic use. The "AGP catalysis” model (B) demonstrates the active induction of resistant bacteria through AGP use. Created with BioRender.com

It is currently understood and widely accepted that the passive emergence of antibiotic-resistant bacteria is an innate outcome of using antibiotics [69],[81],[82]. The increased selective pressure for resistance genes/mechanisms on bacteria from being in the presence of antibiotics seems to follow the basic biological principle of “survival of the fittest” and increases proportionally with the overall volume of antibiotics being used (not necessarily concentration) [83]. This is the lens through which academia, industry, agriculture and the public have viewed the generation of AMR bacteria since the inception of antibiotics (Figure 1a).

Advances in our understanding of bacterial physiology, stress mechanisms and metabolic states allow for the construction of a new framework to understand the effect of using low-dose antibiotics more accurately in agriculture. As stated in the previous section, stressful environments, like those containing elevated levels of reactive oxygen species, low nutrients or antibiotics, activate bacterial stress responses and tend to result in lower metabolism and lower overall activity until the stressor is alleviated [45],[48],[84]. This is also the case for sub-MICs of antibiotics which induce a persister-like phenotype in bacteria. This is seen in slower overall growth, decreased motility and decreased biofilm formation, among other phenotypes [85],[86]. An increase in the persister phenotype has been positively correlated to an increase in the emergence of resistant bacteria [11],[14]. This model indicates that the use of subtherapeutic doses of antibiotics to promote the growth of livestock is catalyzing the emergence of antibiotic-resistant bacteria to a greater degree than was previously assumed using the simplistic “survival of the fittest” framework for AMR generation (Figure 1b).

This catalysis is especially concerning considering which antibiotics are being used as AGPs. While the following antibiotics are not all used to promote growth, they are all used in an agricultural setting for prophylactic purposes or to treat already established infections in animals, thus contributing to the catalysis of AMR bacteria. Medically, tetracyclines are used to treat certain respiratory infections like pneumonia, and infections of the skin, eyes, intestinal, lymphatic, genital and urinary systems. It is also used to treat certain infections spread by insects like lice, mites and ticks [87]. Beta-lactams, which include penicillin, are used to treat numerous types of infections caused by both gram-positive and gram-negative bacteria like syphilis, endocarditis, sinusitis, pneumonia, meningitis, urinary tract infections (UTIs), gonorrhea and severe Lyme disease [88]. Quinolones are commonly used in adults to treat UTIs, pyelonephritis, sexually transmitted infections, prostatitis, gastrointestinal and intraabdominal infections. [89]. The antibiotics used in agriculture have many other medical uses. The continued use of medically important antibiotics in agriculture is rapidly leading to the impotence of these drugs through the previously proposed catalysis model of AMR generation. Certain actions can be taken to lessen or eliminate this debilitating effect on antibiotics.

### 3.2. Countermeasures to the catalysis of AMR bacteria

**Figure 2:**
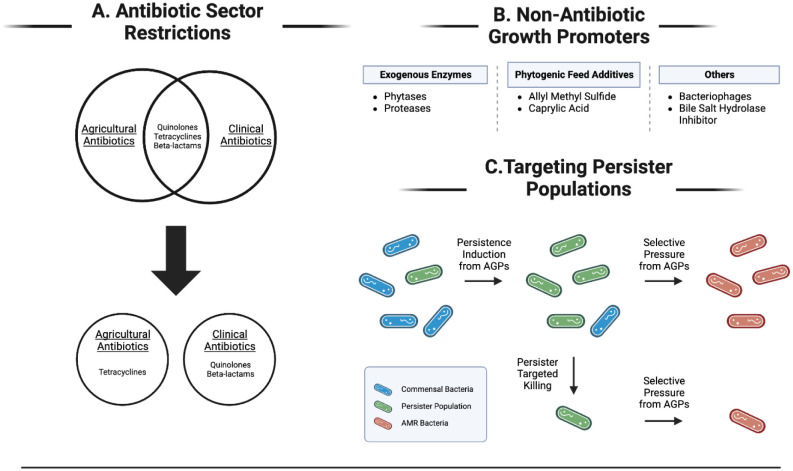
Countermeasures against the catalysis of antibiotic-resistant bacteria by AGPs. (A) The restriction of certain classes of antibiotics to specific sectors would reduce the overlap of commonly used antibiotics and decrease future recalcitrant diseases. (B) The development and implementation of nonantibiotic growth promoters would decrease selective pressure for resistant bacteria [90],[91]. (C) Specific killing of persister populations would decrease the time and quantity of bacteria that AGPs can exert selective pressure on, resulting in a decreased emergence of resistant bacteria. Created with BioRender.com

Three actions can be taken to lengthen the feasible use of medically important antibiotics: (1) restricting agriculturally used antibiotics and minimizing the overlap between these antibiotics and medically important antibiotics (Figure 2a), (2) developing and using other non-antibiotic growth promoters in agriculture (Figure 2b) and (3) specifically targeting persister cells to reduce the emergence of AMR bacteria (Figure 2c).

One potential method to increase the longevity of antibiotic usefulness is to assign a few select classes of antibiotics, like tetracyclines, to strictly agricultural use and allow the remaining antibiotic classes to be used in a strictly clinical setting, thus decreasing the amount of overlap between agricultural and clinical antibiotics (Figure 2a). While this would potentially hurt the output and increase the amount of feed needed to maintain a similar level of protein production due to decreased variety in available antibiotics, it would also decrease future recalcitrant infections in humans that could be caused by AGP-catalyzed AMR bacteria.

A second potential way to increase the functional lifespan of medically relevant antibiotics is to invest in the development and use of nonantibiotic growth promoters (NAGP) (Figure 2b). While the overall efficiency of NAGPs would initially be lower than conventional AGPs, enough time and resources could result in NAGPs matching or bypassing the effects of their predecessors. A few examples of NAGPs that have been proposed include exogenous enzymes like phytases and proteases [90], bacteriophages, phytogenic feed additives like allyl methyl sulfide or caprylic acid and bile salt hydrolase inhibitors [91].

The third potential way to increase the amount of time that medically important antibiotics remain relevant is to selectively target persister cell populations to stop the emergence of novel AMR bacteria (Figure 2c). One way to accomplish this task is to use membrane-targeting antimicrobial peptides (AMPs) that do not rely on the metabolic and energetic state of the cell for their mechanism of action. Examples of these include colistin and daptomycin [92]. A secondary method is to treat these populations with compounds that activate bacterial metabolism, thus suppressing the persistence phenotype and increasing their vulnerability to antibiotics. Some examples of these compounds include glucose, mannitol, fructose and adenosine which potentiate antibiotic killing with gentamycin (for the first three compounds) [52] and gentamycin, ciprofloxacin, ampicillin and ceftriaxone (for adenosine) [75]. While this method could reduce the overall persister population, the physiological effects of all the aforementioned compounds have not been tested regarding in vivo models.

While using any of these methods may negatively affect agriculture, doing so would increase the amount of time that medically important antibiotics that serve a dual function as AGPs could remain in use to treat previously deadly infections. This additional time could also be used to develop novel antibiotics or alternative antimicrobial compounds for clinical use.

In addition to these countermeasures, a fourth action, outside of the scope of this review, would be to ban the use of AGPs. While this would result in the desired effect of decreasing AGP-catalyzed AMR emergence, the overall side effects of this ban would need to be heavily considered before taking action, as it has been in other countries [93]–[97]. Certain countries have already taken steps in banning the use of AGPs either outright, like Sweden in 1986, the European Union in 2006, or China in 2020 (except herbal medicine), or in an antibiotic-specific manner, like Canada or the United States only banning medically important antibiotics from AGP use [98]. In 2013, the United States Food and Drug Administration (US FDA) published *Guidance for Industry #213* (GFI #213) [99] which mandated that the use of medically important antibiotics in food-producing animals must be under the supervision of a licensed veterinarian. Consequentially, as shown in the US FDA 2021 *Summary Report on Antimicrobials Sold or Distributed for Use in Food-Producing Animals*, the total over-the-counter and veterinarian-supervised use of medically important antibiotics peaked in 2015 at 9,702,943 kg [100]. After full implementation of GFI #213, while use of medically important antibiotics in animal feed has been eliminated, “therapeutic” use of medically important antibiotics under veterinarian supervision has more than doubled, and the net decrease in use of medically important antibiotics in food-producing animals has only decreased by 33%. Moreover, according to the 2021 report, while use of medically important antibiotics purely for production purposes has been eliminated, 64% of these antibiotics are used under a veterinary feed directive, which requires veterinarian supervision for treatment of a specific condition. Over the same period, the use of non-medically-important antibiotics has decreased by 11%. While these policies are a step in the right direction, the use of medically important antibiotics in food-producing animals is widespread. One consequence of these policies is that the lines between therapeutic and production uses of antibiotics have likely been blurred, making it difficult now to estimate the contribution of AMR emergence in agriculture from therapeutic clinical indications compared to production purposes.

The feasibility of banning AGPs was demonstrated in a study conducted by Li et al. in 2022 [101]. They analyzed the amount and resistance of *Salmonella* in 47 farms, split between farms that use conventional feed (CF) containing AGPs and farms that previously used AGPs but now use antibiotic-free feed (AFF). They found that samples taken from AFF farms had a lower occurrence of *Salmonella* (2.68% of samples compared to 8.29% in CF farms). Additionally, *Salmonella* isolated from AFF farms tended to possess a higher degree of multi-drug resistance than CF isolates. However, Li et al. suggest that these isolates existed before an AFF mode was adopted. This data suggests that the concentration of *Salmonella* serovars are positively related to the use of AGPs.

## 4. Conclusion

The use of medically important antibiotics in food-producing animals, whether as AGPs or therapeutically for the treatment of zoonotic infectious diseases, is widespread. Numerous studies spanning decades throughout the world have shown that these practices are associated with the emergence of AMR. We have highlighted that the use of AGPs expands bacterial persister populations in food-producing animals and thus accelerates the development of AMR. Although studies are largely correlational, high rates of AMR in bacterial pathogens derived from food-producing animals correlate with higher rates of AMR in pathogens infecting humans, demonstrating a clear threat to public health.

Though the use of AGPs has been banned in many parts of the world, antibiotic usage data still shows liberal use of medically important antibiotics in food producing animals. Ongoing research to guide policy decisions will be crucial to slow the rate at which antibiotic use in food producing animals drives AMR emergence. Here, we have proposed that AGPs catalyze the emergence of AMR. While all the individual parts of the “AGP catalysis” hypothesis have been experimentally verified–use of AGPs leading to increased resistance, antibiotics inducing the persister phenotype and increased persistence leading to increased resistance–the hypothesis itself still requires experimentation [7],[14],[84]. Further research into these mechanisms may provide insight into interventions that can be used to combat AMR while preserving agricultural productivity.
